# Tuning the performance of the non-fullerene organic solar cells by the polarizability

**DOI:** 10.1039/c7ra11297a

**Published:** 2018-01-19

**Authors:** Manman Li, Yuancheng Qin, Weili Dai, Xubiao Luo

**Affiliations:** Key Laboratory of Jiangxi Province for Persistent Pollutants Control and Resources Recycle, Nanchang Hangkong University Nanchang 330063 PR China qinyuancheng@hotmail.com luoxubiao@126.com

## Abstract

We report here the synthesis and characterizations of a novel series of acceptor copolymers with a broad absorption band. The acceptor polymers were synthesized as a copolymer of perylenediimide (PDI) and naphthalene imide (NDI) along with dithieno[3,2-*b*:2′,3′-*d*]silole (DTS) and *N*-alkyl dithieno[3,2-*b*:2′,3-*d*]pyrroles (DTP). When the dipole moment and polarizability of the acceptor polymer are compared, it is observed that when the dipole moment decreases, the polarizability becomes larger. The polarizability of polymers containing PDI is significantly greater than those containing NDI, and their polarizability change is in accordance with the change in the transient fluorescence lifetime. It was also found that the power conversion efficiency of the non-fullerene solar cell was strongly correlated to polarizability. The results demonstrate that the polarizability can be utilized to screen novel donor and acceptor polymers for the design and synthesis of high-performance solar cells.

## Abstract 2

The PCE of solar cells has a direct relationship with the polarizability is also directly related to the PL lifetime. The performance of non-fullerene solar cells can be controlled by polarizability.

## Graphical abstract

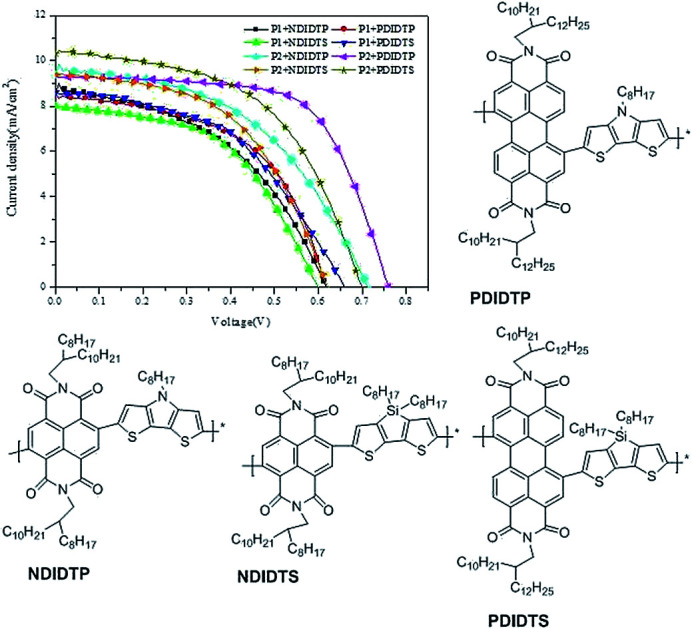

## 1. Introduction

Organic solar cells are a new type of energy device that uses solar energy to generate electricity, and they have generated great interest because of their low cost, light weight, low pollution emission, and the ease with which large panels can be manufactured.^1–3^ Bulk heterojunction polymer solar cells contain polymer donors and fullerene acceptors, and they have now reached 9–11% high power conversion efficiency (PCE).^4–8^ Despite the success of high-efficiency organic solar cells in the laboratory, they are not conducive to practical application because they are expensive and modification of fullerenes is difficult, and therefore, they are not the most rational organic solar cell candidate. In contrast, non-fullerene acceptor materials are low cost, have wide and strong absorption, are easy to adjust with regard to energy levels and design synthesis, possess good mechanical properties, good thermal, optical, and chemical stability and long-term stability, and have generated more interest in the past few years.^9–12^ Currently, several excellent polymer/non-fullerene solar cell systems have a power conversion efficiency (PCE) of more than 13%.^13^ Based on the rapid development of non-fullerene-acceptor organic solar cells, it is speculated that non-fullerene materials will be more promising than fullerene acceptor materials and will exhibit more potential for use in organic solar cells.

Although the PCE of non-fullerene organic solar cells currently does not achieve a high commercial value, researchers have been continuously tuning the performance of non-fullerene solar cells. For example, performance has been increased with the all-polymer solar cell that exhibits a high thermal stability of the PCE up to 11%;^14^ additionally, gains have been made from the study of strong π–π stacking effects of the wide bandgap of non-fullerene polymer,^15^ by adjusting the structure of non-fullerene acceptor polymer,^16^ adjusting the polymer molecular weight,^17^ and regulating the molecular orientation of the polymer.^18^ Therefore, many factors can influence the optical and electrical properties of organic photovoltaic devices. Carsten *et al.*^19,20^ has been studying the effect of the dipole moment on the charge separation in the active layer, and therefore, the influence of the polarizability of non-fullerene acceptor polymers on the performance of solar cell is examined in this study.

In the donor and acceptor polymers, the shape of the electron cloud changes, so that there is an electronic cloud bias to the side of the molecule, resulting in polarization, and the polarizability is the degree of polarization measurement. Polarization is the dipole moment caused by the change in the shape of the electron cloud.^19–21^ Therefore, the internal dipole moment has a strong influence on charge separation in the conjugated polymer.^22^

In this article, perylenediimide (PDI) and naphthalene imide (NDI) were used as electron-donating conjugated polymers, and dithieno[3,2-*b*:2′,3′-*d*]silole (DTS) and *N*-alkyl dithieno[3,2-*b*:2′,3-*d*]pyrroles (DTP) were used as D–A conjugated polymer electron-withdrawing units to synthesize the polymer. Calculating the dipole moment change of the repeating polymer units helps us to identify the different acceptor polymer systems and to determine the effect of the polarizability of the different acceptor polymers on the performance of the photovoltaic device.^23–25^ PDI has the advantages of high thermal stability, wide absorption intensity, and the ability to match the donor energy level. It also has excellent properties of the electron acceptor and high electron mobility, and therefore, it is widely used in the synthesis of non-fullerene electron acceptor materials.^26–28^ However, the strong aggregation effect of PDI molecules is not conducive to improving solar cell performance. Compared with PDI, NDI has a similar structure, but its bandgap is wide, and therefore, it is difficult for the spectral absorption to reach the visible range.^29^

In the polymer, dithieno[3,2-*b*:2′,3′-*d*]silole (DTS) and *N*-alkyl dithieno[3,2-*b*:2′,3-*d*]pyrroles (DTP) are used as the electron-withdrawing units of the D–A conjugated polymer, which are strong in electron-withdrawing ability and have π–π stacking ability, in order to overcome the strong aggregation of the donating electron to form a good electron transport copolymer. Therefore, we used donor polymers and acceptor polymers containing PDI and NDI groups to prepare non-fullerene solar cell devices to study the relationship between the polarizability and dipole moment, and the relationship between polarizability and electrons and PCE.

## 2. Experimental

### 2.1. Materials and instruments

Nuclear magnetic resonance (NMR) was used for characterization (Bruker ARX 400). Thermal stability was measured by thermogravimetric analysis (TGA) with a TA Instruments DSC SDT Q600 thermogravimetric analyzer using a N_2_ flow of 100 mL per minute and heat rate at 10 °C per minute. The photocurrent–voltage (*J*–*V*) of the solar cell devices was tested under simulated solar irradiation (Xe Lamp Oriel Sol3A™ Class AAA Solar Simulators 94023A, USA).

### 2.2. Material design and synthesis

**Scheme 1:**
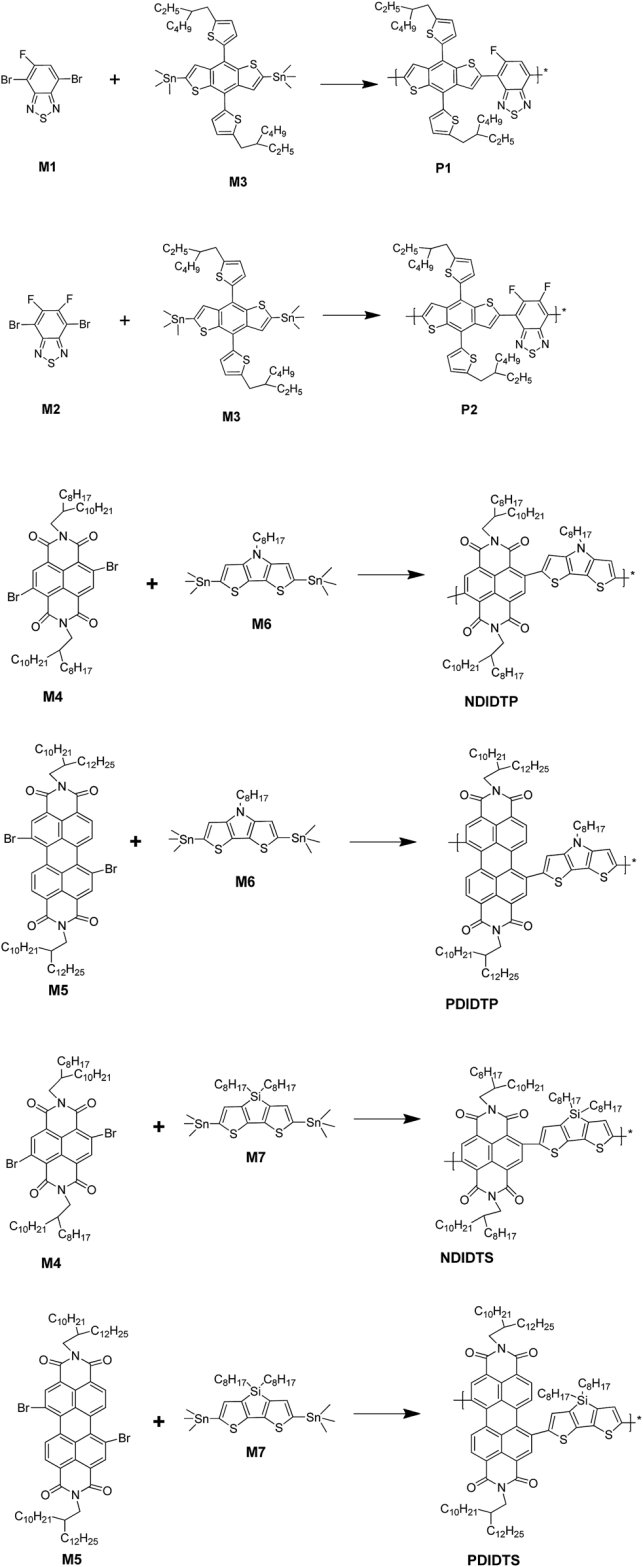
Synthetic route for polymers.

The synthetic route for polymers is shown in Scheme 1.

#### Synthesis of copolymers

Monomer M1 and monomer M3 were added to a double-neck round-bottom flask. Then, the catalyst Pd_2_(dba)_3_, P(*o*-toly)_3_, CuO and 15 mL toluene were added to the solution in the flask. Finally, the solution of monomers was heated for 110 °C and 72 hours under N_2_ protection.

##### P1

M1 (0.3423 g, 1.097 mmol), M3 (0.9925 g, 1.097 mmol), toluene (15 mL), Pd_2_(dba)_3_ (32.598 mg), P(*o*-toly)_3_ (43.228 mg) and CuO (282.28 mg) were used, resulting in a yield of 79% reaction product.

##### P2

M2 (0.268 g, 0.8125 mmol), M3 (0.7349 g, 0.8125 mmol), toluene (15 mL), Pd_2_(dba)_3_ (24.144 mg), P(*o*-toly)_3_ (32.014 mg) and CuO (209.056 mg) were used, resulting in a yield of 81% reaction product.

##### NDIDTP

M4 (0.2182 g, 0.2215 mmol), M6 (0.1366 g, 0.2215 mmol), toluene (10 mL), Pd_2_(dba)_3_ (6.581 mg), P(*o*-toly)_3_ (8.727 mg) and CuO (56.992 mg) were used, resulting in a yield of 70% reaction product.

##### NDIDTS

M4 (0.2364 g, 0.24 mmol), M7 (0.1786 g, 0.24 mmol), toluene (10 mL), Pd_2_(dba)_3_ (7.131 mg), P(*o*-toly)_3_ (9.457 mg) and CuO (61.734 mg) were used, resulting in a yield of 85% reaction product.

##### PDIDTP

M5 (0.2619 g, 0.2145 mmol), M6 (0.1323 g, 0.2145 mmol), toluene (10 mL), Pd_2_(dba)_3_ (6.373 mg), P(*o*-toly)_3_ (8.451 mg) and CuO (55.185 mg) were used, resulting in a yield of 87% reaction product.

##### PDIDTS

M5 (0.2490 g, 0.2038 mmol), M7 (0.1517 g, 0.2038 mmol), toluene (10 mL), Pd_2_(dba)_3_ (6.056 mg), P(*o*-toly)_3_ (8.027 mg) and CuO (52.419 mg) were used, resulting in a yield of 78% reaction product.

### 2.3. Device fabrication

The ITO glass was cleaned with soapy water, deionized water, ethanol, and then acetone using ultrasonication for 15 minutes at every step. The ZnO solution was spin-coated at 3000 rpm and annealed at 220 °C for 30 minutes to prepare for the electron transport layer. The active layer solution (D/A = 1 : 1) was completely dissolved in chlorobenzene, with subsequent heating and stirring at 60 °C overnight. The active layer was spin-coated in a glovebox at 1000 rpm and annealed at 110 °C for 10 minutes. Then, a 7 nm thick layer of MoO_3_ was deposited by evaporation on the blend film in a vacuum evaporation box. Finally, the top 100 nm thick Al electrode layer was deposited by evaporation under the same conditions. The effective region of the device was 8 mm^2^. Each sample was used to fabricate 30 devices.

## 3. Results and discussion

### 3.1. Thermogravimetric analysis

**Fig. 1:**
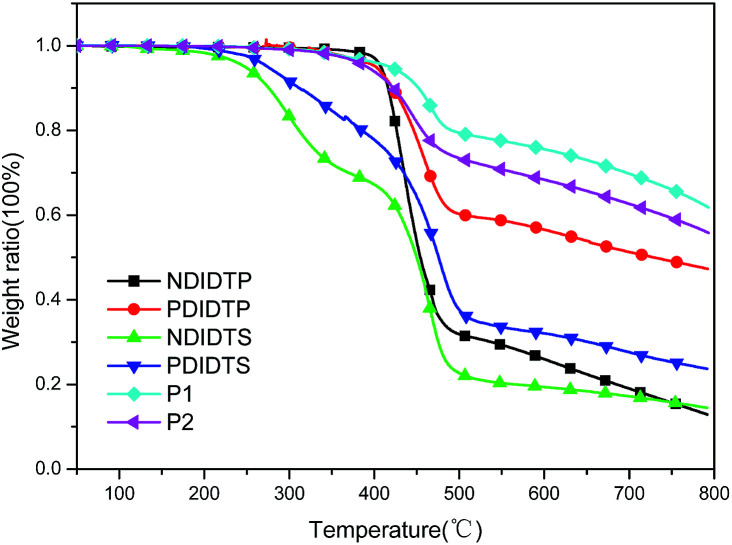
TGA curves of polymers.

**Fig. 2:**
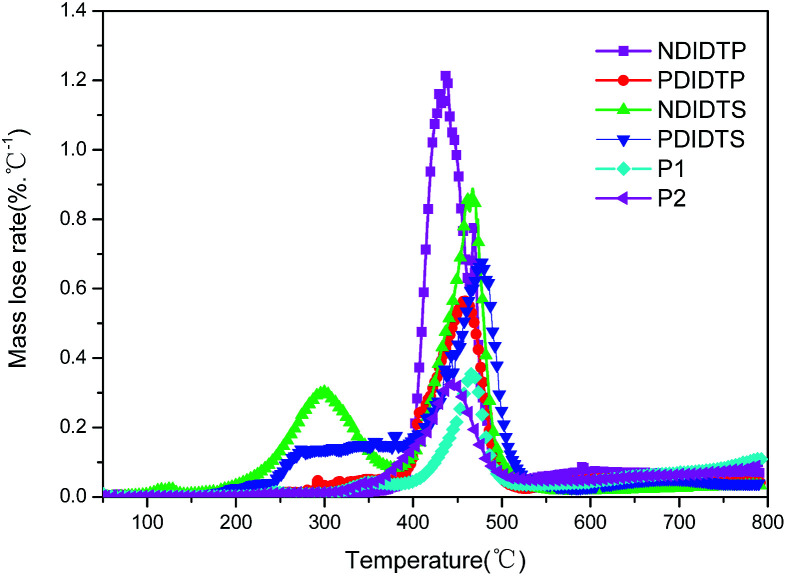
The mass heat loss curves of polymers.

The TGA curves show that all polymers have excellent thermal stability and meet the preparation conditions of the device. From the curve in the Fig. 1, it can be concluded that the thermal stability of P2-1 and P2-2 is significantly better than that of P2-3 and P2-4. P2-3 and P2-4 have a two-stage decomposition characteristic, and the first stage decomposition temperature is lower, which is probably due to the easy decomposition of the DTS group in the polymer at 200–400 °C, thus forming a second peak on the derivative thermogravimetric analysis curve (Fig. 2). According to the thermal decomposition curve, the 5% thermal weightlessness of the four acceptor polymers (P2-1, P2-2, P2-3, and P2-4) was 407 °C, 401 °C, 247 °C, and 274 °C, respectively, and the two donor polymers (P1 and P2) were 418 °C and 391 °C, respectively. Therefore, it can be concluded from the figure that the polymer containing the DTP group is more stable than the polymer containing the DTS group, and its decomposition temperature is higher. In the mass heat loss rate curve, the decomposition weightlessness peak temperature (DWPT) is the anti-degradation capability of the polymer, indicating that the four acceptor polymers have good anti-degradation ability. Therefore, as shown in Table 1, the initial weightlessness temperature of polymer P2-1 is the highest, and the initial decomposition temperature of the polymer with the DTS group is significantly reduced.

**Table 1:** The thermal decomposition characteristics of polymers^a^

Polymer	*T*_i_/°C	*T*_5%_/°C	DWPT/°C	DWPV/% °C^−1^
P2-1	356	407	436	1.21
P2-2	316	401	458	0.56
P2-3	164	247	298	0.30
P2-4	213	274	310	0.14
P1	316	418	466	0.35
P2	301	391	444	0.32

a DWPT = decomposition weightlessness peak temperature; DWPV = decomposition weightlessness peak velocity.

### 3.2. Absorption properties

**Fig. 3:**
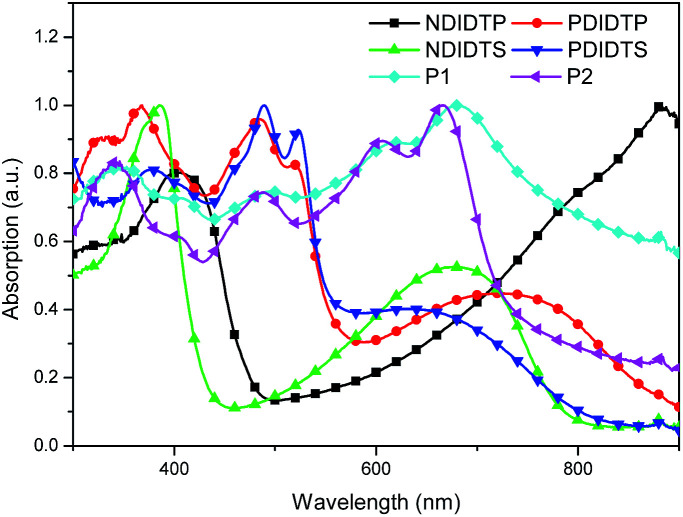
Normalized UV-Vis absorption spectra of the polymers in solution.

The optical absorption spectra of the six polymers are shown in Fig. 3. As shown in Fig. 3, the maximum absorption peak of P1 was at 680 nm, and the maximum absorption peak of P2 was at 665 nm. Therefore, compared with P1, P2 has a clear blueshift, which is explained by the fact that P2 introduces a fluorine atom with greater frequency than that of P1, and in the range of 300 nm to 700 nm, the two polymers have a plurality of absorption peaks. Compared with P2-1, P2-3 has an obvious blueshift in the 300 nm to 500 nm. At 300 nm to 550 nm, the P2-2 has a distinct blueshift compared with P2-4. It can be seen distinctly in Fig. 3 that P2-1 is higher than the absorption of other three acceptor polymers at 900 nm. Therefore, the absorption of the donor polymer and the acceptor polymer is complementary to each other and can cover the entire absorption spectrum range. A wide spectrum range and absorption in the near infrared range are the high performance guarantees for a non-fullerene solar cell.

### 3.3. Fluorescence lifetime

**Fig. 4:**
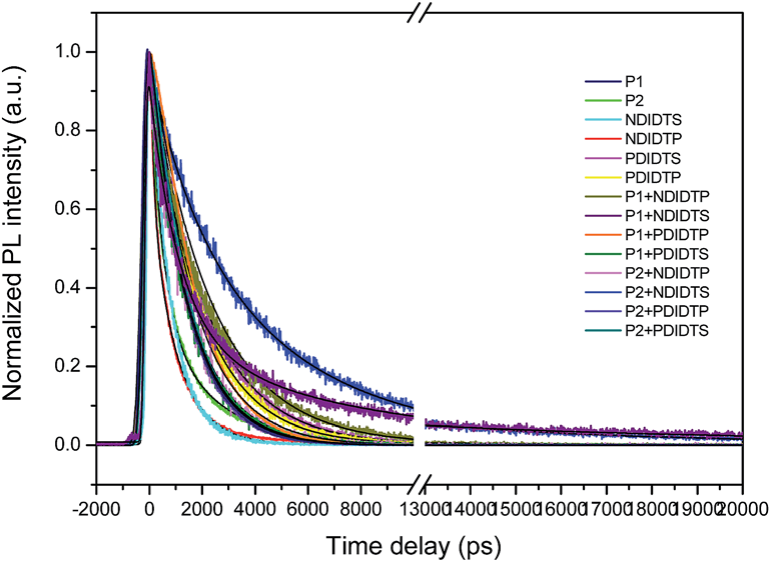
Time-resolved PL results of the polymers.

The photoluminescence (PL) lifetime of organic photovoltaic materials plays an important role in ensuring that the photoexcited excitons have a sufficiently long lifetime to reach the donor and acceptor surfaces. We were pleasantly surprised to find that the degree of the decay of photoluminescence lifetime in all non-fullerene acceptor materials was consistent with the degree of change in the PCE. The transient fluorescence lifetime was calculated by the exponential decay kinetic fitting formula:
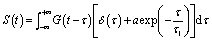
where *S* is the signal, *G* is the laser pulse autocorrelation function, *a* is the amplitude, and *τ* is a time constant.^30–32^ The exponential decay can be well fitted to the attenuation (Fig. 4), and the kinetic index is given in Table 2.

**Table 2:** Kinetic parameters obtained from fits to the PL decays

Kinetic parameters	*a*	*τ*_1_ (ns)
P1	0.95	2.5
P2	1.08	1.5
NDIDTP	0.97	0.8
PDIDTP	0.98	4.4
NDIDTS	1.01	1.4
PDIDTS	1.07	4.4
P1 + NDIDTP	1.09	4.5
P2 + NDIDTP	0.93	1.8
P1 + PDIDTP	0.96	4.4
P2 + PDIDTP	1.07	4.3
P1 + NDIDTS	1.00	4.6
P2 + NDIDTS	1.05	4.8
P1 + PDIDTS	0.98	4.3
P2 + PDIDTS	1.08	4.2

The acceptor polymers containing the PDI group have longer transient fluorescence lifetimes than polymers from the NDI group. Because the PDI derivatives contain strong fluorescent compounds, they have photochemical stability, large Stokes shifts, and good photophysical properties. Moreover, the PDI derivatives possess a large push–pull electronic conjugated system that is vulnerable to irradiation and transitions so as to produce strong fluorescence. In addition, PDI is a π-electron conjugated system with a rigid planar structure. With the increase of the π-electron-conjugated degree and the molecular planarity, the effective π-electron nonlocality is greater. Thus, the acceptor polymer containing the PDI group has a longer transient fluorescence lifetime than that of the NDI group. It can be seen from the table that the transient fluorescence lifetime significantly increases when the donor and acceptor polymer blend, which is due to the electron transition from excited singlet state to the triplet state because the lifetime of the triplet exciton is longer than that of the single exciton. Moreover, the electron relaxation transition between the polymer gaps leads to the transfer of electron energy, resulting in a longer transient fluorescence lifetime.

### 3.4. Polarizability

In the physical sense of the polymer, the dipole moment represents the polarity of the molecule, and the ability to attract electrons is different due to the different constituent elements in the molecule, which creates the phenomenon of electron shift in the molecule that produces polarity.^33^ The greater the dipole moment is, the greater the polarity of the molecule. In addition, the molecular structure can be approximately regarded as an electron cloud and a molecular skeleton composition. Through the determination of the dipole moments, it can be understood that the distribution of the electron clouds and symmetry of the molecule in the molecular structure can be used to identify the geometric isomers and the stereostructure of the molecule.^34^ The data from the dipole moment in Table 3 is comparable to the solar cell performance and shows that the low-dipole donor polymer has a higher performance than the bulk heterojunction with the acceptor polymer.

**Table 3:** Dipole moment and polarizability of polymers

Polymer	P1	P2	NDIDTP	PDIDTP	NDIDTS	PDIDTS
Dipole moment	2.1659	0.3369	6.2836	4.3792	5.9026	3.3000
Polarizability	2.1058	2.1269	2.2929	2.8911	2.2856	2.8589

In addition, in two different atoms, due to the different electron attraction of atomic nuclei, when they combine together, the electron cloud of the entire molecule will tend to move to one side, resulting in polarization, and the polarizability is the degree of polarization measurement. Because PDI has a large π–π conjugated molecular structure, its π electrons are delocalized in the molecule, and the polarizability of the π electron cloud is very high, and therefore, the polymer with a PDI group in the acceptor polymer has higher polarizability. Table 3 also shows that the polarizability is large when the dipole moment is small in donor polymers, and in acceptor polymers, the polarizability first increases and then decreases when the dipole moment increases. It also can be inferred that when the dipole moment is high, the calculated polarizability is low. However, the variation of polarizability is similar to the transient fluorescence lifetime, which occurs because the polarizability and intermolecular forces are mainly related to the dispersion force and induction force. Therefore, the greater the polarizability, the greater the dispersion force and induction force with other molecules, and the greater the polarizability, the longer the transient fluorescence lifetime.

### 3.5. *J*–*V* characterization

**Fig. 5:**
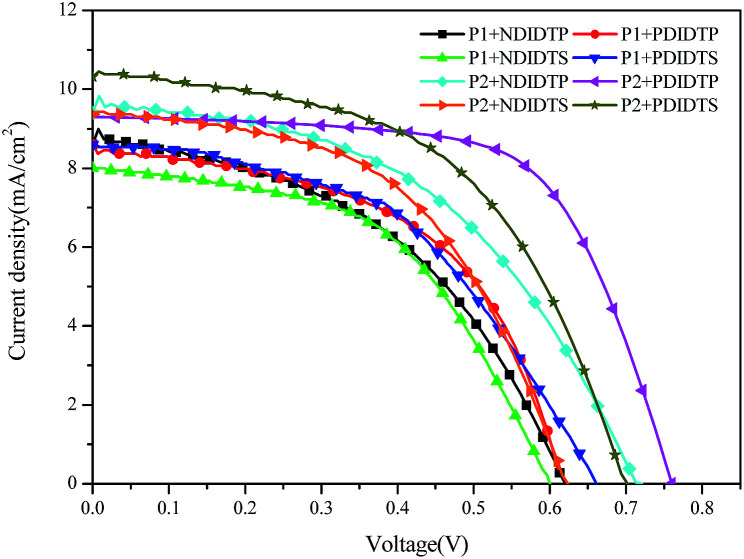
*J*–*V* characteristic curves of a non-fullerene solar cell.

**Fig. 6:**
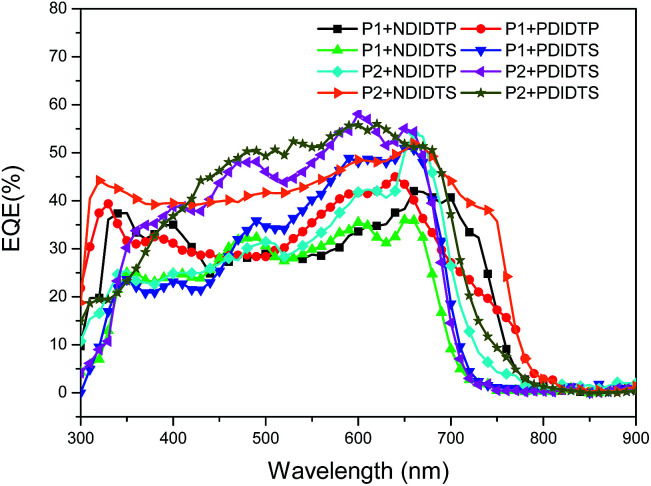
EQE curves of a non-fullerene solar cell.

The parameters of the solar cell are indicated in Table 4. It can be seen from the table that the PCE of the P2 polymer containing fluorine atoms is higher than that containing P1 polymer. By comparing the polarizability with the PCE data, it was found that the PCE increases with increasing polarizability when polymer P2 binds to the acceptor (Fig. 5). When P1 and the receptor bind, the PCE increases with the polarization rate and then decreases. Therefore, it is likely that when the polarizability is a certain value, the PCE will attain the maximum value. This will be the next direction for our research. The acceptor polymer containing the PDI group has a higher PCE than the polymer solar cell containing the NDI group. Moreover, Fig. 6 shows the external quantum efficiency (EQE) of the non-fullerene solar cell. Compared with the UV-Vis absorption spectra, the EQE result was in general accord with the UV-Vis absorption spectra. However, unlike the UV-Vis absorption spectra, the EQE of the solar cell containing NDIDTP acceptor polymer is not very high or pronounced in near-infrared. The non-fullerene solar cell containing P2 polymer led to better change split and transport, which was obtained by enhanced EQE and *J*_sc_ in devices. This is caused by the effect of fluorine atoms. The incorporation of fluorine atoms in the donor polymer has a strong effect on intermolecular and intermolecular interactions, and it can regulate the energy level of the polymer. Fluorine atoms have strong electronegativity, which results in a strong electron-withdrawing effect, and polymers containing fluorine atoms have a higher PCE. In addition, the PDI group contains a large benzene ring planar structure and two imine ring structures, with high electron affinity and strong electron capture ability. PDI has a large π–π conjugated structure, its π electrons are delocalized in the molecule, and the π electron cloud has high polarizability; therefore, the interaction of π electron clouds may cause π electrons to form a delocalization belt between molecules, and then promote the conjugation of the π electron structure between the organic molecules, as well as the electric conductivity and photoelectric conversion capacity.

**Table 4:** Performance of a non-fullerene solar cell

Polymer	*V*_oc_ (mV)	*J*_sc_ (mA cm^−2^)	FF (%)	PCE (%)
P1 + NDIDTP	0.62 ± 0.01	8.6 ± 0.3	50.2 ± 1	2.68 ± 0.02
P1 + PDIDTP	0.61 ± 0.01	8.56 ± 0.2	54.0 ± 1	2.82 ± 0.03
P1 + NDIDTS	0.60 ± 0.01	8.0 ± 0.2	51.0 ± 2	2.45 ± 0.03
P1 + PDIDTS	0.66 ± 0.01	8.59 ± 0.3	52.0 ± 2	2.95 ± 0.03
P2 + NDIDTP	0.72 ± 0.01	9.4 ± 0.3	52.2 ± 2	3.53 ± 0.03
P2 + PDIDTP	0.76 ± 0.01	9.3 ± 0.2	57.3 ± 1	4.05 ± 0.02
P2 + NDIDTS	0.62 ± 0.01	9.4 ± 0.3	55.1 ± 1	3.21 ± 0.02
P2 + PDIDTS	0.7 ± 0.01	10.3 ± 0.2	54.1 ± 1	3.90 ± 0.03

## 4. Conclusions

Four acceptor polymers and two donor polymers were used in preparing the non-fullerene solar cell. It was found that the PCE of the solar cell has a direct relationship with the polarizability, and the polarizability is also directly related to the transient fluorescence lifetime. In this study, for the calculated results of polymer dipole moment and polarizability, it was determined that the polarizability is large when the dipole moment is small in donor polymers, and in acceptor polymers, the polarizability first increases and then decreases when the dipole moment increases. When the high polarizability donor polymer is combined with the non-fullerene acceptor, its PCE increases with increasing polarizability. When the low polarizability donor polymer is combined with the non-fullerene acceptor, its PCE increases first and then decreases with the increase in polarizability. Therefore, when the donor and acceptor are combined, the large polarizability indicates that the electron cloud migration is enhanced and the interaction between the positive and negative ions is reduced, which reduces the Coulomb binding energy between the acceptor and the electron, and increases the electron mobility. Furthermore, it is likely that when the polarizability is a certain value, the PCE will have the maximum value. This will be the next direction that deserves our research. In addition, the polarizability of acceptors containing PDI is larger than that containing NDI, and the transient fluorescence lifetime is also long, mainly because PDI has a higher electron mobility than NDI. Thus, the large polarizability of the polymer increases the excitons separated into electrons and holes, thereby reducing the overall Coulomb binding energy and increasing the electron mobility. Although the photovoltaic effect of the bulk heterojunction solar cell is a complex phenomenon, we find that the polarizability can be used to screen different donor and acceptor polymers for the design and synthesis of high performance solar cells.

## Conflicts of interest

There are no conflicts of interest to declare.

## Supplementary Material

## References

[cit1] Zhong Y., Trinh M. T., Chen R., Wang W., Khlyabich P. P., Kumar B., Sfeir M. Y., Black C., Steigerwald M. L., Loo Y.-L., Xiao S., Ng F., Zhu X. Y., Nuckolls C. (2014). J. Am. Chem. Soc..

[cit2] Green M. A., Emery K., Hishikawa Y., Warta W., Dunlop E. D. (2015). Prog. Photovoltaics Res. Appl..

[cit3] Lee J., Jo S. B., Kim M., Kim H. G., Shin J., Kim H., Cho K. (2014). Adv. Mater..

[cit4] Chuang C.-H. M., Brown P. R., Bulović V., Bawendi M. G. (2014). Nat. Mater..

[cit5] He Z., Xiao B., Liu F., Wu H., Yang Y., Xiao S., Wang C., Russell T. P., Cao Y. (2015). Nat. Photonics.

[cit6] Kan B., Li M., Zhang Q., Liu F., Wan X., Wang Y., Ni W., Long G., Yang X., Feng H., Zuo Y., Zhang M., Huang F., Cao Y., Russell T. P., Chen Y. (2015). J. Am. Chem. Soc..

[cit7] Liu Y., Zhao J., Li Z., Mu C., Ma W., Hu H., Jiang K., Lin H., Ade H., Yan H. (2014). Nat. Commun..

[cit8] Zhang G., Zhang K., Yin Q., Jiang X. F., Wang Z., Xin J., Ma W., Yan H., Huang F., Cao Y. (2017). J. Am. Chem. Soc..

[cit9] Ye L., Zhao W., Li S., Mukherjee S., Carpenter J. H., Awartani O., Jiao X., Hou J., Ade H. (2017). Adv. Energy Mater..

[cit10] Cha H., Wu J., Wadsworth A., Nagitta J., Limbu S., Pont S., Li Z., Searle J., Wyatt M. F., Baran D., Kim J.-S., McCulloch I., Durrant J. R. (2017). Adv. Mater..

[cit11] Zhao F., Dai S., Wu Y., Zhang Q., Wang J., Jiang L., Ling Q., Wei Z., Ma W., You W., Wang C., Zhan X. (2017). Adv. Mater..

[cit12] Zuo L., Yu J., Shi X., Lin F., Tang W., Jen K. Y. (2017). Adv. Mater..

[cit13] Yong C., Yao H., Gao B., Qin Y., Zhang S., Bei Y., Chang H., Xu B., Hou J. (2017). J. Am. Chem. Soc..

[cit14] Zhao W., Qian D., Zhang S., Li S., Inganäs O., Gao F., Hou J. (2016). Adv. Mater..

[cit15] Yao H., Yu R., Shin T. J., Zhang H., Zhang S., Jang B., Uddin M. A., Woo H. Y., Hou J. (2016). Adv. Energy Mater..

[cit16] Nielsen C. B., Holliday S., Chen H. Y., Cryer S., Mcculloch I. (2015). Acc. Chem. Res..

[cit17] Zhou N., Dudnik A. S., Li T. I., Manley E. F., Aldrich T. J., Guo P., Liao H. C., Chen Z., Chen L. X., Chang R. P. (2016). J. Am. Chem. Soc..

[cit18] Jung J., Lee W., Lee C., Ahn H., Kim B. J. (2016). Adv. Energy Mater..

[cit19] Carsten B., Szarko J. M., Lu L., Son H. J., He F., Botros Y. Y., Chen L. X., Yu L. (2012). Macromolecules.

[cit20] Carsten B., Szarko J. M., Son H. J., Wang W., Lu L., He F., Rolczynski B. S., Lou S. J., Chen L. X., Yu L. (2011). J. Am. Chem. Soc..

[cit21] Xu T., Lu L., Zheng T., Szarko J. M., Schneider A., Chen L. X., Yu L. (2014). Adv. Funct. Mater..

[cit22] Intemann J. J., Yao K., Ding F., Xu Y., Xin X., Li X., Jen A. K. Y. (2015). Adv. Funct. Mater..

[cit23] Wu Q., Zhao D., Schneider A. M., Chen W., Yu L. (2016). J. Am. Chem. Soc..

[cit24] Zhao D., Wu Q., Cai Z., Zheng T., Chen W., Lu J., Yu L. (2016). Chem. Mater..

[cit25] Duan Y., Xu X., He Y., Wu W., Li Z., Peng Q. (2017). Adv. Mater..

[cit26] McAfee S. M., Dayneko S. V., Hendsbee A. D., Josse P., Blanchard P., Cabanetos C., Welch G. C. (2017). J. Mater. Chem. A.

[cit27] Wang Z., Zheng N., Zhang W., Yan H., Xie Z., Ma Y., Huang F., Cao Y. (2017). Adv. Energy Mater..

[cit28] Xiao B., Tang A., Zhang J., Mahmood A., Wei Z., Zhou E. (2017). Adv. Energy Mater..

[cit29] Rundel K., Maniam S., Deshmukh K., Gann E., Prasad S. K. K., Hodgkiss J. M., Langford S. J., McNeill C. R. (2017). J. Mater. Chem. A.

[cit30] Li Q., Jin X., Yang Y., Wang H., Xu H., Cheng Y., Wei T., Qin Y., Luo X., Sun W. (2016). Adv. Funct. Mater..

[cit31] Li Q., Jin X., Song Y., Zhang Q., Xu Z., Chen Z., Cheng Y., Luo X. (2015). ACS Appl. Mater. Interfaces.

[cit32] Li M., Qin Y., Yan C., Dai W., Luo X., Jin X., Li Q. (2016). Synth. Met..

[cit33] Würfel U., Seßler M., Unmüssig M., Hofmann N., List M., Mankel E., Mayer T., Reiter G., Bubendorff J. L., Simon L. (2016). Adv. Energy Mater..

[cit34] Takacs C. J., Sun Y., Welch G. C., Perez L. A., Liu X., Wen W., Bazan G. C., Heeger A. J. (2012). J. Am. Chem. Soc..

